# Sleep Quality and Aging: A Systematic Review on Healthy Older People, Mild Cognitive Impairment and Alzheimer’s Disease

**DOI:** 10.3390/ijerph19148457

**Published:** 2022-07-11

**Authors:** Maria Casagrande, Giuseppe Forte, Francesca Favieri, Ilaria Corbo

**Affiliations:** 1 Department of Dynamic and Clinical Psychology and Health Studies, Sapienza University of Rome, 00185 Roma, Italy; g.forte@uniroma1.it; 2 Body and Action Laboratory, IRCCS Santa Lucia Foundation, Via Ardeatina 306, 00179 Rome, Italy; francesca.favieri@uniroma1.it; 3 Department of Psychology, Sapienza University of Rome, Via dei Marsi 78, 00185 Roma, Italy

**Keywords:** sleep quality, mild cognitive impairment, Alzheimer, aging, healthy elderly, older

## Abstract

Aging is characterized by changes in the structure and quality of sleep. When the alterations in sleep become substantial, they can generate or accelerate cognitive decline, even in the absence of overt pathology. In fact, impaired sleep represents one of the earliest symptoms of Alzheimer’s disease (AD). This systematic review aimed to analyze the studies on sleep quality in aging, also considering mild cognitive impairment (MCI) and AD. The review process was conducted according to the PRISMA statement. A total of 71 studies were included, and the whole sample had a mean age that ranged from 58.3 to 93.7 years (62.8–93.7 healthy participants and 61.8–86.7 pathological populations). Of these selected studies, 33 adopt subjective measurements, 31 adopt objective measures, and 10 studies used both. Pathological aging showed a worse impoverishment of sleep than older adults, in both subjective and objective measurements. The most common aspect compromised in AD and MCI were REM sleep, sleep efficiency, sleep latency, and sleep duration. These results underline that sleep alterations are associated with cognitive impairment. In conclusion, the frequency and severity of sleep disturbance appear to follow the evolution of cognitive impairment. The overall results of objective measures seem more consistent than those highlighted by subjective measurements.

## 1. Introduction

Aging is associated with a physiological decline that also involves cognitive domains. In clinical conditions, such as mild cognitive impairment (MCI) or dementia, cognitive decline is altered compared to physiological ones [1,2]. Dementia currently affects more than 55 million people, and this figure is estimated to rise to 78 million in 2030 [3]. Alzheimer’s disease (AD) accounts for the majority of dementia cases [4]. For these reasons, the early detection of possible precursors of dementia and the diagnosis and treatment of modifiable risk factors are increasing in importance.

In recent years, evaluation of prodromal risk-state for dementia, such as MCI [5], has become relevant for its impact on older people’s quality of life. MCI is classified into two subtypes: amnestic (i.e., clinically significant memory impairment) and non-amnestic (i.e., decline in cognitive functions unrelated to memory). Moreover, other cognitive domains are considered and often compromised, allowing to classify MCI into single-domain amnestic/non-amnestic and multiple-domain amnestic/non-amnestic types [5,6]. To date, no reliable treatments are available for dementia. For these reasons, maintaining the well-being of people also in a prodromal state is an increasing priority, as well as identifying risk factors for cognitive impairment. In this sense, considering sleep quality and duration and sleep disorders can be relevant.

Sleep disturbances are characterized by a decreased sleep duration and quality, reduced sleep efficiency, increased sleep fragmentation, and diurnal sleepiness. Sleep disturbances are common in aging and there is an increase in sleep disorders in pathological aging [7,8,9,10,11,12]. Different studies showed that aging-related poor sleep quality is associated with a worsening in cognitive functions, e.g., [10,13,14], specifically associated with excessive diurnal sleepiness and attentional and executive impairments [15,16,17] as well as general cognitive impairment.

Sleep quality directly affects daily activities and is involved in individual psychological, cognitive, and physical well-being, e.g., [18,19]. Moreover, a cumulative index of sleep problems, rather than specific symptoms of poor sleep, represents the biggest risk factor for cognitive impairment [10]. However, some studies have not highlighted an association between sleep disturbance and impaired cognitive functioning [20]; on the contrary, other authors have reported higher cognitive functioning in a large sample of participants with insomnia [21].

Sleep disorders are common in AD and are involved in memory consolidation impairment [22] and metabolite removal from synapses (i.e., including β-amyloid), which are implicated in the neurogenesis of AD [22,23]. Sleep may also play an important role in cognitive reserve [24] and restoring neurobehavioral functions and psychological aspects, e.g., [25].

One out of four individuals with AD exhibits severe sleep dysfunctions, such as repetitive awakenings or other sleep disorders, i.e., insomnia, hypersomnia, or circadian rhythm disturbances [26,27,28]. Some authors identified sleep disorders in the preclinical phases of AD as a predictor of dementia incidence, such as in MCI [11,29,30,31,32,33,34,35,36]. A third of MCI people exhibit sleep disorders [29]. Most commonly reported sleep disorders are insomnia, sleep breathing-related disorders, restless legs syndrome, and REM sleep behavior disorders [7,32]. However, these results are inconsistent, with some studies reporting no association between sleep disturbances and cognitive decline [20].

This systematic review aims to clarify the relationship between sleep quality and aging, providing the main characteristics of sleep and sleep disturbances in the continuum from healthy aging to MCI and AD. Moreover, results are analyzed according to the type of measure—subjective or objective—used to assess sleep characteristics.

Specifically, this systematic review aims to: (1) evaluate the sleep characteristics of people with MCI compared to healthy older adults; (2) assess the sleep characteristics in people diagnosed with AD compared to healthy older adults; (3) evaluate the sleep characteristics in people diagnosed with MCI compared to AD; (4) compare the presence and severity of sleep disturbances among AD, MCI, and healthy subjects; (5) compare the differences between healthy, MCI and AD subjects in sleepiness, that is considered one of the more predominant features of poor sleep and sleep disorders; (6) assess whether poor sleep quality can be considered a reliable marker of cognitive impairment in a continuum from healthy older adult to mild cognitive impairment to Alzheimer disease.

Given that sleep disorders in the preclinical phases of AD are predictors of dementia incidence [7,29,30,31,32,33,34,35,36], we should find poorer sleep in people diagnosed with MCI or AD than in healthy older people. Furthermore, sleep should be worse, and sleep disturbances should be higher in AD than in MCI people.

## 2. Materials and Methods

The review process was conducted according to the PRISMA statement [37,38].

### 2.1. Research Strategies

Two independent researchers (IC, GF) consulted four electronic bibliographic database searches (PsychINFO, PubMed, Medline, PsycArticles). A list of keywords and MeSH terms was generated to identify studies (MCI OR Mild Cognitive Impairment OR dementia OR Alzheimer OR AD); and (sleep); and (elderly OR aged OR older OR elder OR geriatric OR elderly people OR old people OR senior). The last search was conducted on 18 December 2021. Restrictions were made, limiting the research to academic publications with English full texts and studies on human populations without restrictions regarding gender and ethnicity. Additionally, the bibliographical references of retrieved papers, reviews, and meta-analyses were screened manually to assess whether they contained relevant studies to include in the review.

Due to the wide variety of instruments used, diagnostic criteria, and data, a meta-analysis could not be performed.

The search strategies are presented in Table 1.

### 2.2. Eligibility Criteria

**Figure 1:**
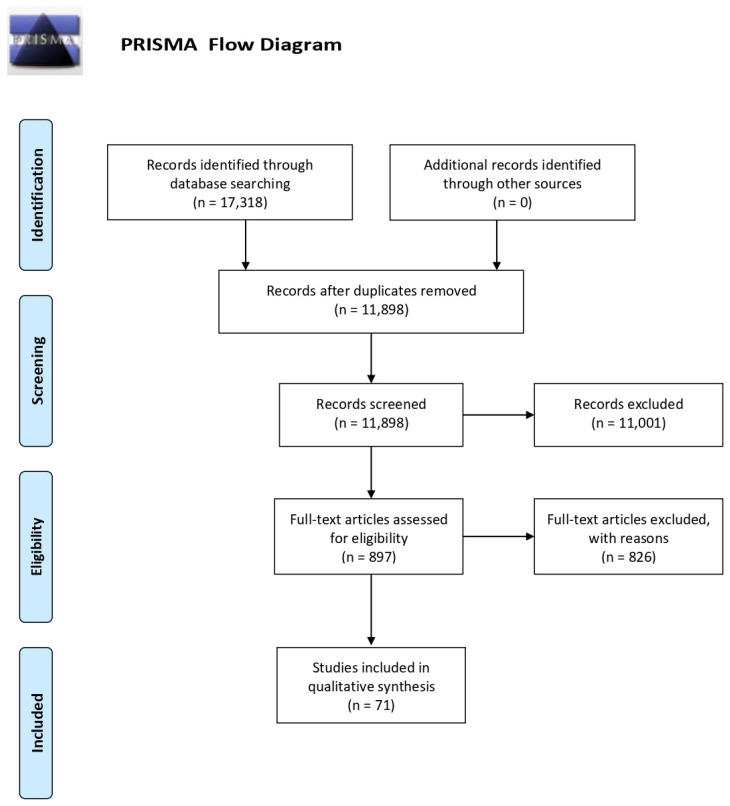
PRISMA flow diagram.

A total of 17,318 articles were obtained from the search procedure. The first step allows 5420 duplicates that were eliminated using the Mendeley software. Then, the list of potential articles produced by systematic research was revised. The reading of the title and abstract allowed the first exclusion of 11,001 studies. A further selection was made by reading the full text (see Figure 1). Two researchers independently performed the eligibility assessment. A supervisor (MC) resolved disagreements. Review and randomized control trials or intervention studies were excluded.

We selected studies that included the adult population (age equal to or higher than 50 years), diagnosis of mild cognitive impairment; diagnosis of Alzheimer’s disease; healthy subjects; subjective sleep measurements; objective sleep measurements (polysomnography and actigraphy).

We enclosed both studies that included participants with a previous formal diagnosis and studies that diagnosed and classified participants based on their performance in a pool of neuropsychological tests.

We excluded studies including participants with (a) medical conditions that could potentially influence the investigated relationship (e.g., metabolic disorders; cardiovascular diseases; chronic conditions; cancer); (b) dementia (Parkinson’s disease; vascular dementia; frontotemporal dementia; dementia with Lewy bodies; Huntington’s disease); (c) psychiatric or neurological disorders; (d) strokes; (e) head traumas; (f) sleep disorders; (g) use of drugs that affect the nervous system or sleep; (h) studies that presented methodological criticisms; (i) studies that do not report essential data or have an assessment made by caregivers; (l) MCI participants included in healthy older people or AD groups.

### 2.3. Data Collection

According to the PICOS approach [38], information was extracted from each included study on (1) author(s) and year of publication; (2) characteristics of participants (including age, gender, Mini-Mental State Examination—MMSE score); (3) diagnostic criteria; (4) experimental paradigm; (5) results of the studies. Data were extracted by two researchers (IC and GF), and other researchers (FF and MC) were involved in case of controversy.

The methodological quality of studies was assessed.

### 2.4. Quality Assessment

The quality of the studies was assessed using the Cochrane Handbook for Systematic Reviews criteria [39], adapted ad hoc according to the objective of this review in order to reduce the risk bias. The analysis used five criteria to screen each study selected for systematic review: sampling bias, sleep measurements, diagnostic criteria, selective reporting bias, and methodological bias. Each criterion score ranges from 1 (low risk) to 3 (high risk). The overall quality shall be calculated by adding all the scores, ranging from 5 to 15. The study was considered at low risk of bias if the score was 5, while a 6–10 range score was considered an indicator of a moderate risk of bias, and an 11–15 range score meant a high risk of bias. The quality assessment was separately made for subjective and objective measurements.

Two independent researchers, IC and GF, rated all articles included in the study, and two other researchers (FF and MC) were involved in case of disputes.

## 3. Results

### 3.1. Studies Selection

The flow chart shows the number of studies identified from the databases, and the number of studies examined, assessed for eligibility, and included in the review with the reasons for possible exclusions (see Figure 1). A total of 71 studies were included in the final analysis. The most common exclusion criteria are: diagnosis of non-Alzheimer dementia, diagnosed sleep disorders (i.e., sleep apnea, insomnia, REM sleep behavior disorder), neurological disorders (i.e., stroke, head injury), and psychiatric disorders (i.e., depression, schizophrenia).

Of the 71 selected studies, 33 adopt subjective measurements (questionnaires, sleep diaries, and interviews), and 31 adopt objective measures (23 polysomnography and 8 actigraphy). Ten studies used both measures (6 a combination of questionnaires and polysomnography; 4 questionnaires and actigraphy).

Seventy of the seventy-one studies (98.6%) used a cross-sectional design.

Results will be presented in two subsections: (1) sleep in older age and (2) sleep in pathological older age, also considering the type of measurements adopted.

### 3.2. Quality Assessment of Subjective Measurements

**Figure 2:**
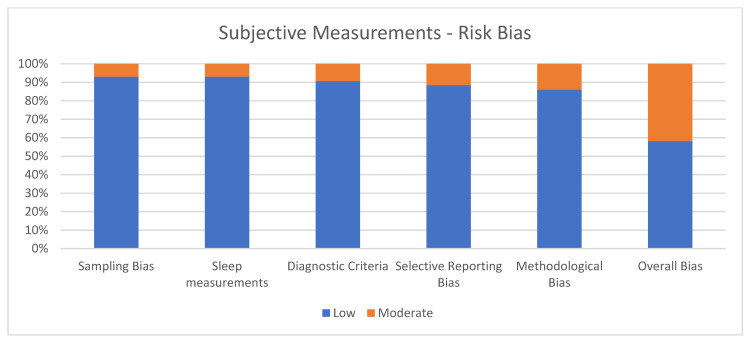
Subjective measurements.

Figure 2 shows the percentage of articles adopting subjective measurements fulfilling each quality criterion assessed by the risk of bias assessment. On average, the quality of the studies was good since 25 out of 43 (58.1%) exhibited a low risk of bias. The high percentage of studies with low or no risk of bias increases the validity of this systematic review. Despite 18 studies (41.9%) showing moderate scores, no study reports a moderate risk of bias in more than two items. A large percentage of the studies adopted valid and reliable tools to measure sleep quality and included an appropriate sample size. Moreover, most studies were adequately controlled for confounding variables. The higher risk of bias was in the “methodological bias” and the lower in “sleep measurements”. The risk of bias ranged from 5 to 7 for every article included (see Figure 2).

### 3.3. Quality Assessment of Objective Measurements

**Figure 3:**
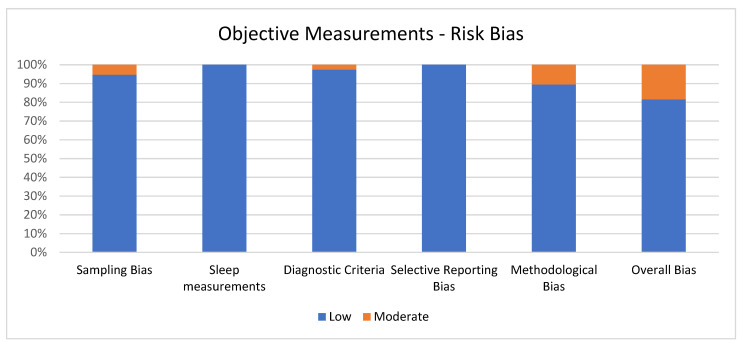
Objective measurements.

Figure 3 shows the percentage of articles adopting objective measurement fulfilling each quality criterion of risk of bias assessment. On average, the quality of the studies was good since 31 out of 38 (81.6%) exhibited low scores on the risk of bias. The high percentage of studies with low or no risk of bias increases the validity of this systematic review. Despite 7 studies (18.4%) showing moderate scores, no study reports a moderate risk of bias in more than one item. A large percentage of the studies used valid and reliable tools to measure sleep quality and included an appropriate sample size. Moreover, most studies were adequately controlled for confounding variables. The higher risk bias was in the “methodological bias” and the lower in “sleep measurements” and “selective reporting bias”. The score ranged from 5 to 6 for every article included for the overall bias (see Figure 3).

### 3.4. Sleep in Healthy Older People

#### 3.4.1. Subjective Measurements (N = 14)

Fourteen studies adopted subjective measures to assess sleep quality in healthy older people, with an overall sample size of 1680 individuals and a mean age ranging from 67.2 [40] to 93.7 years [41]. The most adopted tools to assess sleep quality and disturbances were the Pittsburgh Sleep Quality Index—PSQI [42] and the Neuropsychiatric Inventory—NPI. The PSQI assesses seven components of sleep (sleep quality, sleep latency, sleep duration, habitual sleep efficiency, sleep disturbance, use of sleep medication, and daytime dysfunctions) and a global score that measures and discriminates “good sleepers” and “poor sleepers”. The NPI is a questionnaire evaluating twelve behavioral disturbances occurring in dementia, including sleep disturbances (i.e., hallucination, dysphoria, delusions, anxiety, agitation/aggression, euphoria, disinhibition, irritability, apathy, aberrant motor activity, eating abnormalities, and sleep disturbance) and measuring frequency and severity of the symptoms.

##### Pittsburgh Sleep Quality Index—PSQI Results (N = 11)

Eleven studies met the inclusion criteria and used the PSQI [42] to evaluate sleep quality in healthy older people [40,41,43,44,45,46,47,48,49,50,51]. Six studies considered the percentage of the sample with poor sleep quality [41,43,44,48,51], showing a poor sleep quality that varies from 11.2% [43] to 64% [48]. Five studies reported the average PSQI global score [40,45,46,47,50] with a range from 3.9 [45] to 6.3 [47]. One study [49] reported sleep duration (443.4 min) and sleep latency (12.5 min) of the participants.

##### Neuropsychiatric Inventory—NPI Results (N = 2)

Two studies adopted the NPI to evaluate sleep quality in healthy older people [52,53], underlining a prevalence of sleep disturbances of 3.8% [53] and 6.5% [52] in the considered samples.

##### Other Subjective Sleep Measurement Results (N = 4)

Two studies collected data about sleep routines through a sleep diary [48,54]. Bliwise et al. [54] analyzed gender differences in sleep habits but did not find significant differences in sleep quality between males and females. Landry et al. [48] reported a sleep latency of 22.6 min, an average number of awakenings of 2.1, and a sleep duration of 398 min in a sample of 78 participants with a mean age of 71.6. One study met the inclusion criteria and used the Insomnia Severity Index (ISI) to evaluate insomnia severity [43], underlining a prevalence of moderate or severe insomnia in 7.8% of participants with a mean age of 69.9. One study met the inclusion criteria and used the “St. Mary’s Hospital Sleep Questionnaire” [49] to evaluate the sleep-related routines, reporting a sleep latency of 12.4 min and sleep duration of 426 min in a sample of 14 participants with a mean age of 69.1.

Table 2 shows the main characteristics of the studies.

#### 3.4.2. Objective Measurements (N = 7)

Seven studies assessed the sleep characteristics in healthy older people, with an overall sample of 216 participants and ages ranging from 62.8 [55] to 82.2 [56].

##### Polysomnographic Results (N = 3)

Three studies met the inclusion criteria and adopted polysomnography to evaluate sleep quality in healthy older people [45,57,58]. The overall sample size was 57 participants, with a mean age ranging from 68.6 [45] to 69 years [57].

The most investigated index was the REM latency, ranging from 57.6 [58] to 91 min [45]. Curcio et al. [45] have compared various polysomnographic variables (total sleep time, total bedtime, sleep efficiency, number of awakenings, percentage, and latency of NREM1, NREM2, REM, and slow-wave sleep) in five different groups: young, healthy older people, subjects with depression, subjects with dementia, and subjects with obstructive sleep apnea syndrome. The healthy older people showed a higher percentage of NREM2 sleep than the other groups.

Prinz et al. [57] compared healthy older people with three other groups (subjects with mild, moderate, and severe dementia), underlining that healthy older people have a better sleep quality than people with dementia, considering time in bed (TIB), NREM 3–4 (%TIB), REM (%TIB), wakefulness (%TIB), number of awakenings, and REM latency (min)].

Reynolds III et al. [58] compared healthy older people with demented and depressed people, considering sleep Latency (min), wakefulness (min), time spent asleep (min), arousal (number), % sleep efficiency, % sleep maintenance, %NREM1, %NREM2, %NREM 3–4, %NREM, %REM, LREM (min), and REM time (min), finding a lower number of sleep-related disorders in healthy older people (see Table 3).

Table 3 shows the main characteristics of the studies.

##### Actigraphy Results (N = 4)

Four studies met the inclusion criteria and used actigraphy to evaluate sleep quality in healthy older people [48,56,59,60]. The overall sample size was 159 participants, with a mean age ranging from 62.8 [55] to 82.2 years [56].

Both Wilckens et al. [59] and Kume et al. [56] reported a total sleep time ranging from 362.4 to 380.2 min. Paavilainen et al. [60] showed that time in bed in healthy older people (540 min) was lower than in people with dementia. Landry et al. [48] reported a 19.5% prevalence of poor sleep quality, evidenced by a composite score based on sleep fragmentation equal to or higher than 40, sleep efficiency equal to or less than 75, or sleep duration equal to or less than 360 min.

Table 4 shows the main characteristics of the studies.

### 3.5. Sleep in Pathological Older People

The systematic review identified 49 studies assessing sleep quality or sleep disturbances in older people with pathological cognitive impairment. The age of the samples ranged from 62.1 [61] to 86.7 years [59]. Twenty-six studies included a sample with a diagnosis of AD [46,61,62,63,64,65,66,67,68,69,70,71,72,73,74,75,76,77,78,79,80,81,82,83,84,85,86], and 28 included a sample with a diagnosis of MCI [63,65,66,68,69,84,87,88,89,90,91,92,93,94,95,96,97,98,99,100,101,102,103,104,105,106,107,108,109,110].

#### 3.5.1. Diagnostic Criteria

Different diagnostic criteria were adopted in the selected studies for the AD or MCI diagnosis.

Twenty studies adopted the diagnostic criteria of the “National Institute of Neurological and Communicative Diseases and Stroke -Alzheimer’s Disease and Related Disorders Association—NINCDS-ADRDA” [46,61,62,64,65,66,67,70,71,72,73,74,77,79,81,83,86,94,101,105,111]. Four studies followed the diagnostic criteria of the “Alzheimer’s Association and the National Institute on Aging—NIA-AA” [63,69,84,85,112]. Three studies used the diagnostic criteria of “Diagnostic and Statistical Manual of Mental Disorders—DSM” ed. III [71,76,80,113]. Three studies adopted the diagnostic criteria of “Diagnostic and Statistical Manual of Mental Disorders—DSM” ed. IV [46,63,93,114]. Two studies followed the diagnostic criteria of “Diagnostic and Statistical Manual of Mental Disorders—DSM” ed. 5 [68,88,115]. Eighteen studies adopted the diagnostic criteria of Petersen [5,63,66,69,87,88,90,92,96,97,99,100,101,102,104,106,110,116,117,118,119]. Five studies used the diagnostic criteria of Albert et al. [59,66,85,92,108,120]. Four studies used the “Mini-Mental State Examination—MMSE” [121] as a diagnostic criterion, with different cut-offs: MMSE lower than 24 [91], MMSE lower than 20 [78], MMSE lower than 21 [80], MMSE lower than 17 [109]. Seven studies adopted other criteria [122,123,124,125], for example, Portet et al. criteria [63,65,75,88,95,98,107,109,122].

#### 3.5.2. Subjective Measurements (N = 27)

Twenty-seven studies assessed sleep quality in pathological older people with a subjective measure. The overall sample size was 4587 participants (540 AD, 1960 MCI, and 2086 healthy older people), with a mean age ranging from 63.7 [90] to 79 years [64].

Nine [62,63,64,65,66,67,68,69,70] studies reported data from AD people and twenty-four [63,65,66,68,69,87,88,89,90,91,92,93,94,95,96,97,98,100,101,102,103,106,107,109] studies on people with MCI diagnosis.

##### Pittsburgh Sleep Quality Index—PSQI Results (N = 11)

Eleven studies used the PSQI [42] to evaluate sleep quality in pathological older people [63,67,68,70,97,98,99,101,102,103,106]. Tuna et al. [98] included subjects with MCI, underlining a 53.6% prevalence of poor sleep quality in the sample. Shin et al. [67] included subjects with AD, highlighting a slightly poor sleep quality (mean score 5.4) in the sample. Two studies compared sleep quality in healthy, MCI, and AD older people [63,68]. Gorgoni et al. [63] did not report significant differences between the groups, while Tadokoro et al. [68] found a poorer sleep quality in AD subjects than in MCI and healthy subjects.

Six studies [97,99,101,102,103,106] compared the PSQI scores between healthy and MCI older people. Only Sun et al. [97] and Yu et al. [99] reported poorer sleep quality in MCI, while the other studies did not highlight any significant differences [101,102,103]. Zhou et al. [70] compared healthy and AD participants, finding higher PSQI scores and a higher prevalence of poor sleep quality (55.9%) in AD people than in healthy participants (15.2%).

##### Neuropsychiatric Inventory—NPI Results (N = 13)

Thirteen studies met the inclusion criteria and used the NPI [42] to evaluate the sleep quality of pathological older people [62,64,65,66,69,70,89,90,91,93,94,95].

Peters et al. [93], Reijs et al. [94], and Rozzini et al. [95] assessed sleep quality in older people with MCI diagnosis. The first two studies reported a prevalence of sleep disturbances of 39.9% and 22%, respectively, while Rozzini et al. [95] did not find evidence of sleep disturbances in older people with MCI (mean score: 0.5). Fernández-Martínez et al. [62] and Matsuoka et al. [64] evaluated the NPI in people with AD diagnosis and reported a prevalence of sleep disturbances of respectively 35.1% and 30.2%. Two studies [87,90] compared the sleep disturbances prevalence in healthy and MCI older people. Fernández-Martínez et al. [87] found significant differences between the two groups in sleep disturbances prevalence (MCI = 23,1% vs. healthy older people = 14%). Similarly, Muangpaisan et al. [90] reported a prevalence of sleep disturbances of 45.5% in MCI and 23.3% in the healthy control group. Ng et al. [91], comparing healthy older people and subjects affected by preclinical AD, showed that older people with preclinical AD reported poorer sleep quality than the control group. Lee et al. [89] investigated the difference in sleep disturbances between aMCI and naMCI, but did not find significant differences (18.8% aMCI, 18% naMCI). Two studies [66,69] compared older people with MCI and AD, underlining a poorer sleep quality in AD. In particular, Yatawara et al. [69] observed a prevalence of sleep disturbances of 31% in MCI and 40% in AD. Zhou et al. [70] compared a healthy group with an AD group, reporting a higher number of sleep disturbances in AD. Pocnet et al. [65], on the other hand, assessed the progression of sleep disturbances in a range of two years in healthy, MCI, and AD subjects. The results showed that sleep disturbances remained stable in healthy and AD older people while increased in MCI subjects.

##### Other Subjective Measurements Results (N = 9)

Sun et al. [97] used the “Insomnia Severity Index—ISI” to evaluate the insomnia severity in healthy and MCI subjects, underlining a greater severity in the MCI group.

One study adopted the “Karolinska Sleep Diary” [102] to evaluate the sleep routine but did not report differences between aMCI and healthy subjects.

One study collected data about sleep routines through an interview [88]. The MCI group showed higher sleep latency, awakenings, sleep after awakening, and lower sleep duration and quality than the healthy group. One study used the “Athens Insomnia Scale—AIS” [107] and reported higher sleep latency, number of sleep disturbances, total sleep time, night awakening, and earlier awakenings in MCI than in healthy participants. One study adopting the “Jenkins Sleep Questionnaire—JSS” [101] did not report any significant difference between aMCI and the healthy control group. One study collected data about sleep routines through an interview [109] and reported a higher sleep duration in older people with MCI than in healthy subjects. Finally, using the “Sleep Continuity Scale in Alzheimer’s Disease—SCADS” [92], no significant difference in sleep routine was observed in healthy and MCI people. However, older people with MCI showed a poor sleep quality in 21.7% of cases, while the prevalence observed in healthy participants was 15.3%. Table 5 shows the main characteristics of the studies.

#### 3.5.3. Objective Measurements (N = 31)

Thirty-one studies assessed sleep quality in pathological older people using objective measures. The overall sample size was 1415 participants (478 AD, 306 MCI, and 631 healthy older people), with a mean age ranging from 58.3 [77] to 86.7 years [59].

##### Polysomnography Results (N = 23)

Participants assessed with polysomnography have a mean age ranging from 58.3 [77] to 76.9 years [79].

Twenty-three studies met the inclusion criteria and used the polysomnography to evaluate sleep quality in pathological older people [46,61,63,71,72,73,74,75,76,77,78,79,80,81,84,86,96,100,103,104,105,106,110].

Three studies [73,75,81] considered only AD. Tsuno et al. [81] assessed sleep latency in AD and subjects with multi-infarction dementia. No significant differences were reported between groups. Kundermann et al. [73] assessed sleep latency in AD, showing a mean time of 50 min. Total sleep time (ranges from 292.8 min [73] to 368.7 min [75]), sleep efficiency % (ranging from 59.5 [73] to 74.7 [75]); REM latency (that varies from 124.1 min [73] to 204.8 min [75]), and intra-sleep wakefulness (ranging from 130.8 min [75] to 136.2 min [73]) were investigated

Three studies [63,84,105] adopt polysomnography in healthy, MCI, and AD older people, and all reported some differences: Maestri et al. [105] reported a higher NREM stage 1 in AD than in MCI and healthy participants, while Gorgoni et al. [63] reported a lower slow-wave sleep percentage in AD than in the other two groups. Liguori et al. [85] analyzed the differences among healthy subjects, older people with MCI, mild AD (mAD), and mild-severe AD (msAD): msAD showed lower total sleep time, sleep efficiency %, REM %, and higher time in bed than the other groups; in addition, msAD had higher NREM1% than MCI and healthy subjects. Mild Alzheimer (mAD) presented higher total sleep time, NREM3%, REM% than msAD but lower than healthy and MCI groups; higher sleep efficiency % than msAD but lower than healthy subjects; higher time in bed than msAD, and higher intra-sleep wakefulness, REM latency and NREM1% than the healthy group. The older people group showed higher total sleep time, NREM3% than AD groups, higher sleep efficiency % than msAD but lower than the healthy older people group, lower time in bed than msAD, higher REM latency and intra-sleep wakefulness than the healthy group, higher REM % than AD group but lower than healthy older people, and higher NREM1% than healthy subjects but lower than AD groups.

Eleven studies [46,61,71,72,74,76,77,78,79,80,86] have compared AD and healthy subjects.

The participants with AD have shown: higher sleep latency [80,86], NREM1 and NREM2 latency [46], NREM sleep percentage [71], NREM1 sleep [74,86,105], REM latency [74,86], higher intra-sleep wakefulness [74,78,86]; other authors found lower NREM3 [74,86,105], slow wave [46,63,78], and REM sleep percentages [61,77,78,86,105], total sleep time [61,74,80,105], delta waves [76], K-complex [77], sleep spindles [77,79], and sleep efficiency [71,72,74,86].

Six studies [96,100,103,104,106,110] have compared MCI and healthy older people.

The MCI subjects have shown: higher REM Latency [106,110], intra-sleep wakefulness [106]; higher arousal index during the slow-wave sleep period [96,104]; REM % [96,100,104,105], slow-wave sleep % [103], total sleep time [110], and sleep efficiency % [110].

Furthermore, Carnicelli et al. [100] have observed that, after 2 years, 61.1% of MCI participants met AD diagnostic criteria. MCI converters showed a lower REM sleep % than MCI and healthy older people.

Table 6 shows the main characteristics of the studies.

##### Actigraphy Results (N = 8)

Participants assessed with actigraphy have a mean age ranging from 61.8 [85] to 86.7 years [59].

Eight studies met the inclusion criteria and used actigraphy to evaluate sleep quality in pathological older people [59,68,82,83,85,101,102,108].

Three studies compared AD and healthy subjects; Lee et al. [83] did not show a significant difference, while Khou et al. [82] reported a higher total sleep time and a higher time in bed in AD. Liguori et al. [84] observed a higher sleep latency and a lower sleep efficiency in AD.

Four studies compared MCI and healthy subjects; two did not show significant differences. One [101] observed some differences in sleep onset and higher variability in MCI, while the other [108] highlighted lower SE% in MCI subjects.

Tadokoro et al. [68] is the only study that compared MCI, AD, and healthy older people. It reported lower REM sleep and higher NREM1 and NREM2 sleep in AD than in MCI and healthy, and lower REM sleep in MCI than in healthy older people.

Table 7 shows the main characteristics of the studies.

### 3.6. Diurnal Sleepiness (N = 10)

Diurnal sleepiness is not a sleep disorder but a strong indirect index of poor sleep. The most widely used test to assess daytime sleepiness is the Epworth Sleepiness Scale—ESS, which evaluates sleepiness in eight different situations and the chances of feeling drowsiness and falling asleep.

#### 3.6.1. Healthy Older People (N = 2)

Two studies used the Epworth Sleepiness Scale (ESS) to evaluate diurnal sleepiness in healthy older people [43,126]. The sample size includes 507 participants, with a mean age ranging from 69.9 [43] to 73.2 years [126].

Bernstein et al. [43] underlined a prevalence of 18% of sleepiness, while Ward et al. [127] reported a mean score of 5.9 in diurnal sleepiness in the respondents.

Table 8 shows the main characteristics of the studies.

#### 3.6.2. Pathological Older People (N = 8)

Eight studies met the inclusion criteria and used the ESS to evaluate the diurnal sleepiness in pathological older people [68,70,88,96,97,101,102,106]. The sample size includes 515 participants (105 AD, 160 MCI, and 250 healthy older people), with a mean age ranging from 66.5 [70] to 75.6 years [102].

Six of these studies [70,88,96,97,101,102,106] compared daytime sleepiness in healthy and MCI subjects, and only two studies [97,106] reported higher diurnal sleepiness in MCI than healthy older people. Zhou et al. [70] observed higher diurnal sleepiness in Alzheimer’s compared to the healthy group. Finally, Tadokoro et al. [68] compared healthy MCI and AD and did not find significant differences.

Table 9 shows the main characteristics of the studies.

## 4. Discussion

Defining possible risks or exacerbating factors for pathological cognitive decline represents an important goal of the current research on aging and dementia. Accordingly, this systematic review aimed to clarify the possible role of sleep as a predictor of cognitive impairment in aging, analyzing sleep quality and sleep disturbances in healthy and pathological aging. The analyzed studies show that, in older people, the prevalence of sleep disturbance ranges from 3.8% [53] to 64% [48], in the MCI it ranges between 18% [89] and 53.6% [98], and finally in individuals with Alzheimer’s disease, the prevalence ranges between 30.2% [64] and 40% [69].

Twenty-six out of twenty-eight (92.8%) studies that used objective measures and compared healthy and pathological old people found worse sleep quality in the latter, while only nine out of twenty-two (40.9%) studies that used subjective measures showed these differences.

The results pointed out that thirty-four out of forty-five studies (75.5%) show a higher prevalence of sleep disturbances in pathological aging (AD and MCI) than in healthy older people. In the data provided by these studies, we can observe an overall poorer sleep quality in pathological older people, particularly a higher compromission in AD people than in MCI and healthy older people. These results underline that sleep alterations are associated with cognitive impairment [128,129,130,131,132,133,134].

The first goal of our systematic review was to evaluate the sleep characteristics of people with mild cognitive impairment compared to healthy older people, and 17 studies out of 23 (73.9%) observed a lower sleep quality in MCI than in healthy subjects. The main differences reported were lower REM%, slow-wave sleep and sleep efficiency and higher sleep latency, NREM1%, and sleep duration; these findings are confirmed by different studies [13,135,136]. Moreover, these results highlighted that sleep is more compromised in MCI participants despite the high prevalence of poor sleep and sleep disturbances in healthy older people (3.8–64%).

The second goal was to investigate the sleep characteristics in people diagnosed with Alzheimer’s disease compared to healthy older people. The more compromised sleep aspects in AD are slow-wave sleep and REM sleep; consequently, people show high sleep fragmentation and poor sleep efficiency. However, conflicting results emerged regarding stage 2 of NREM sleep [71,105]. In particular, Maestri et al. [105] observed that AD had a higher percentage of stage 2 NREM sleep than healthy older people, while Hoch et al. [71] reported that AD had a lower percentage of stage 2 NREM sleep than healthy older people. Additionally, polysomnographic results show a worsening of sleep, involving both sleep structure, e.g., [46,73,77,78,79,105] and sleep quality [66,68,70,84], in AD than in healthy older people.

The third goal was to assess the differences in the presence and severity of sleep disturbances between MCI and AD. People with AD were compared to older people with MCI in seven studies, and four studies out of seven (57.1%) found poorer sleep quality in AD. In particular, AD exhibited higher NREM1% and time in bed and lower total sleep time, sleep efficiency, NREM3%, and REM% compared to MCI.

The fourth goal was to compare the presence and severity of sleep disturbances among AD, MCI, and healthy subjects. However, only four studies compared these three groups concurrently [63,68,84,105]. Maestri et al. [105] observed that older people with MCI presented intermediate sleep disorders between healthy subjects and people with AD. In fact, MCI had poorer sleep quality than healthy subjects but higher than older people with AD; in addition, Tadokoro et al. [68] observed lower REM sleep percentage and higher percentages of stage 1 and stage 2 NREM sleep in AD than in MCI and healthy subjects, and lower REM sleep in MCI than in healthy older people. While Liguori et al. [85] observed that older people with MCI had lower sleep efficiency, REM% than healthy subjects but higher than subjects with AD. Furthermore, older people with MCI had higher NREM1% than control groups but lower than participants with AD. These results are in line with previous studies analyzing sleep in normal and pathological older age, e.g., [7,8,9,12,28].

The fifth and final goal was to evaluate the differences between healthy, MCI and AD subjects in daytime sleepiness, considered one of the more predominant features of poor sleep and sleep disorders. Only two out of eight studies (25%) found higher sleepiness in subjects with AD and MCI than in healthy older people. The fact that 75% of the studies found no significant difference between the groups can be explained by the increased diurnal sleepiness in the healthy older people control group compared to younger subjects [137,138,139].

Finally, most of the studies were cross-sectional (70 out of 71; 98.6%); therefore, it is impossible to indicate a causal relationship. However, the only longitudinal study (1 out of 71; 1.4%) observed that MCI participants showed a greater increase in sleep disturbances after two years compared to healthy older people and people with AD [65]. Furthermore, other studies [90,140] observed that poor sleep quality was associated with deposits of beta-amyloid plaques and lower volumes in the amygdala, hippocampi, and bilateral parietal lobules compared. These findings agree with results showing that beta-amyloid plaques and hippocampi atrophies seem to be predictors of dementia [140]. According to these data, we can infer that sleep tends to worsen with age until it develops into overt sleep disturbances as MCI and AD progress [7,29,30,32,33,34]. Additionally, the present systematic review results strengthen the hypothesis that a decrease in sleep time and quality, including sleep disturbances, constitutes a strong biological marker of cognitive impairment in aging and for the transition from healthy aging to MCI and from the latter to AD. These results are consistent with well-known data suggesting that sleep loss can lead to cognitive decline [15,16,141,142]. The future direction of this study involves implementing a meta-analysis to also have a quantitative estimate of sleep problems. we also plan to study other dementia conditions, as studying only these two pathological conditions is a limiting factor.

### Limitation

Although the encouraging results, this review holds some limitations.

Despite the considerable number of studies that assessed sleep subjects with AD, just a few studies evaluated sleep in mild cognitive impairment or the influence of poor sleep quality on the cognitive functions of subjects with MCI. The small number of studies on older people with MCI, the multiplicity of instruments used to assess sleep, and the different criteria for diagnosing MCI and AD limited this review, particularly the subjective measures, making comparisons difficult. Another limiting factor was the lack of a meta-analysis, which was difficult to conduct due to the large variety of instruments, diagnostic criteria, and reported results from the examined studies. Another limiting aspect is the lack of longitudinal studies. An additional limiting factor is the presence of multiple types of dementia (mixed dementia) that overlap making it difficult to identify a specific sleep pattern for each dementia phenotype. Lastly, this review exclusively analyzed two types of pathological aging (mild cognitive impairment and Alzheimer’s disease), omitting the other types of dementia (e.g., vascular dementia, frontotemporal dementia).

## 5. Conclusions

This systematic review has shown a higher prevalence of sleep disturbances in older people with mild cognitive impairment and Alzheimer’s disease than in healthy older people. AD and MCI showed modifications over every aspect of sleep: sleep quality, sleep structure, sleep disturbance, and duration of wakefulness intra-sleep. This study could be useful because it tried to investigate the differences between three groups of participants (subjects with AD, MCI, and healthy older people) and highlights the different sleep disturbances found in healthy and pathological aging.

An important goal for the next studies will be to figure out whether sleep disturbances and poor sleep quality are early symptoms of dementia or whether dementia leads to poorer sleep due to the more significant alterations of the nervous system occurring in pathologically older age than in healthy older people.

## Figures and Tables

**Table 1:** Research Strategies.

Database	N°
PsychINFO	2192
PsycArticles	15
Medline	4633
PubMed	10,478

**Table 2:** Sleep in older age. Summary of all the studies that use subjective measures.

Authors	Group	N°	Age (SD)	(%F)	MMSE (SD)	Measurements	Results
Bernstein et al. [43]	HS	423	69.9 (7.6)	64.4	29.5	PSQI ISI	PSQI 11.2% poor sleep quality. ISI 7.8% mild or severe insomnia.
Bliwise et al. [54]	HS	30	73.8	66.7	≥28	Sleep Diary	No difference between males and females.
Brown et al. [44]	HS	184	75.5 (6.1)	58.7	28.9 (1.3)	PSQI	41% poor sleep quality.
Curcio et al. [45]	HS	10	68.6 (7.0)	50	26.7 (1.3)	PSQI	PSQI TOT: 3.9 (0.9)
De Gennaro et al. [46]	HS	20	70.3	60	28.75	PSQI	PSQI TOT: 6.0 (0.7)
Fjell et al. [47]	HS	91	72	51.6	29	PSQI	PSQI TOT: 6.3 (3.8)
Jirong et al. [41]	HS	251	93.7 (3.4)	49.8	-	PSQI	37% poor sleep quality.
Landry et al. [48]	HS	78	71.6 (6.6)	67	28.8 (1.2)	PSQI Sleep Diary	PSQI 64% poor sleep quality. Sleep Diary SL: 22.6 (16.2) Awakenings: 2.1 (1) Sleep windows (min): 461.7 (50.2) SD (min): 398 (61.7)
Mary et al. [49]	HS	14	69.1 (1.5)	57.1	Mattis: 141.4 (1.9)	PSQI St. Mary’s Hospital Sleep Questionnaire	PSQI SD (hr): 7.39 (1.0) SL (min): 12.5 (16.3) SMHSQ SD (hr): 7.1 (1.1) SL (min): 12.4 (12.3)
Rainey-Smith et al. [50]	HS	462	75 (6)	58.1	28.9 (1.3)	PSQI	49.1% poor sleep quality
Sani et al. [51]	HS	25	67.3 (7.5)	60	29.5 (0.7)	PSQI	28% sleep disturbances. 12% excessive diurnal sleepiness. 20% disruptive sleep events.
Schmidt et al. [40]	HS	14	67.2 (4.0)	57.1	Mattis: 139.4 (3.8)	PSQI	PSQI TOT: 3.9 (2.0)
Squelard et al. [52]	HS	46	79.3 (7.9)	76.1	24.7 (6.0)	NPI	6.5% sleep disturbances.
Tatsch et al. [53]	HS	78	72.3 (8.2)	67.9	25.2 (3.6)	NPI	3.8% sleep disturbances.

SD = standard deviation; F = female; ES = standard error; HS = healthy subject; MCI = mild cognitive impairment; aMCI = amnestic mild cognitive impairment; naMCI = non-amnestic mild cognitive impairment; Pre-AD = preclinical Alzheimer’s disease; AD = Alzheimer’s disease; MMSE = Mini Mental State Examination; MoCA = Montreal Cognitive Assessment; CDR = Clinical Dementia Rating Scale; RCS = Rapid Cognitive Screen; T1,2 = Time 1 and Time 2; DSM = Diagnostic and Statistical Manual of Mental Disorders; NIA–AA = National Institute on Aging–Alzheimer’s Association; NINCDS = National Institute of Neurological and Communicative Diseases and Stroke; NINCDS–ADRDA = National Institute of Neurological and Communicative Diseases and Stroke–Alzheimer’s Disease and Related Disorders Association; PSQI = Pittsburgh Sleep Quality Index; NPI = Neuropsychiatric Inventory; ESS = Epworth Sleepiness Scale; ISI = Insomnia Severity Index; AIS = Athens Insomnia Scale; JSS = Jenkins Sleep Questionnaire; SCADS = Sleep Continuity Scale in Alzheimer’s Disease; SMHSQ = St. Mary’s Hospital Sleep Questionnaire; TST = total sleep time; TIB = time in bed; SL = sleep latency; SD = sleep duration; SE = sleep efficiency.

**Table 3:** Sleep in older age. Summary of all the studies that use polysomnography.

Authors	Group	N°	Age (SD)	(%F)	MMSE (SD)	Measurements	Results
Curcio et al. [45]	HS	10	68.6 (7.0)	50	26.7 (1.3)	PSG	L NREM1(min): 18.0 (4.4) L NREM2(min): 21.1 (16.4) L SWS (min): 81.9 (6.5) L REM (min): 146.7 (32.1) NREM1 (%): 12.6 (2.3) NREM2 (%): 69.9 (2.0) SWS (%): 0.4 (0.2) REM (%): 17.0 (1.6) Awakenings (n): 25.0 (2.5) TST (min): 347.5 (22.3) TBT (min): 532.9 (171.7) SE (%): 70.8 (6.4)
Prinz et al. [57]	HS	22	69.0 (6.4)	50	29.6	PSG	TIB: 456.3 (37.7) NREM 3–4 (%TIB): 8.9 (5.2) REM (%TIB): 16.8 (4.1) W (%TIB): 21 (7.2) Awakenings (n°): 13.6 (4.7) LREM: 65 (24.7)
Reynolds III et al. [58]	HS	25	69.0 (5.0)	68	29.2 (0.9)	PSG	SL (min): 22.2 (16.9) W (min): 47.4 (32.8) TSA (min): 368.7 (44.5) Arousal (n): 6.8 (2.7) SE (%): 84.1 (8.3) Sleep maintenance (%): 88.6 (7.3) NREM1(%): 9.7 (8.2) NREM2(%): 64.9 (8.3) Stage N (%): 2.6 (6.2) NREM3–4(%): 2.5 (3.9) REM (%): 20.2 (4.9) LREM: 57.6 (16.9) REM time (min): 75.0 (21.2)

SD = standard deviation; F = female; HS = healthy subject; MMSE = Mini Mental State Examination; PSG = polysomnography; TST = total sleep time; TSA = time spent asleep; TIB = time in bed; TBT = total bed time; SL = sleep latency; SE = sleep efficiency; LNREM = nonREM latency; NREM = nonREM; LREM = REM latency; REM = rapid eye movement; LSWS = slow-wave sleep latency; SWS = sleep wave sleep; WASO = wake after sleep onset; W = wakefulness.

**Table 4:** Sleep in older age. Summary of all the studies that use actigraphy.

Authors	Group	N°	Age (SD)	(%F)	MMSE (SD)	Measurements	Results
Kume et al. [56]	HS	17	82.2 (4.2)	76.4	CDR: 0	Actigraphy	TST (min): 380.2 (43.7) SL: 13.1 (13.5) SE (%): 83.7 (4.1) Awakening time (min): 57.5 (20.7)
Landry et al. [48]	HS	78	71.6 (6.6)	67	28.8 (1.2)	Actigraphy	19.5% poor sleep quality.
Paavilainen et al. [60]	HS	19	81.5 (9.0)	-	26.2 (2.9)	Actigraphy	TIB (h): 9.0 (1.2)
Wilckens et al. 2018 [59]	HS	45	62.8 (6.0)	71.1	28.8 (1.0)	Actigraphy	TST (min): 362.4 (63.1) WASO (min): 63.4 (42.5)

SD = standard deviation; F = female; HS = healthy subject; MMSE = Mini Mental State Examination; CDR = Clinical Dementia Rating Scale; TST = total sleep time; TIB = time in bed; SL = sleep latency; SE = sleep efficiency; WASO = wake after sleep onset.

**Table 5:** Sleep in pathological older age. Summary of all the studies that use subjective measures.

Authors	Group	N°	Age (SD)	(%F)	MMSE (SD)	Diagnostic Criteria	Measurements	Results
Fernández-Martínez et al. [62]	AD	37	74.4 (6.7)	64.9	18.4 (2.8)	NINCDS–ADRDA	NPI	35.1% sleep disturbances.
Fernández-Martínez et al. [87]	MCI HS	91 50	74.2 (5.3) 74.5 (7.0)	45.1 66.0	26.4 (1.8) 28.6 (1.1)	Petersen	NPI	MCI: 23.1% sleep disturbances. HS: 14% sleep disturbances.
Gorgoni et al. [63]	AD aMCI HS	15 15 15	70.8 71.1 70.8	66.7 60.0 33.3	16.1 26.1 29.1	DSM IV Petersen	PSQI	No difference has been found.
Hita-Yañez et al. [88]	HS MCI	25 25	67.1 (5.3) 70.5 (6.8)	52 28	28.1 (1.3) 26.7 (2.4)	DSM V Petersen CDR ≤ 0.5	Interview	MCI: higher WASO. SL and awakenings. MCI: lower sleep quality and SD.
Lee et al. [89]	aMCI naMCI	217 165	72.8 (6.9) 71.5 (5.8)	62.2 69.9	20.8 (4.5) 22.1 (3.8)	Petersen	NPI	aMCI: 18.8% sleep disturbances. naMCI: 18% sleep disturbances. No difference between aMCI and naMCI has been found.
Matsuoka et al. [64]	AD	63	79 (6.9)	69.8	20.1 (4.2)	NINCDS–ADRDA	NPI	30.2% sleep disturbances.
Muangpaisan et al. [90]	MCI HS	77 30	66.3 (7.9) 63.7 (7.3)	35 25	26.5 (1.0) 28.1 (1.8)	Petersen	NPI	MCI: 45.5% sleep disturbances. HS: 23.3% sleep disturbances. No difference between HS and MCI has been found.
Naismith et al. [106]	HS MCI	26 26	65.9 (9.8) 70.1 (9.9)	53.8 34.6	29.2 (1.1) 27.5 (2.1)	Petersen	PSQI	No difference between HS and MCI has been found.
Ng et al. [91]	HS Pre-AD	22 33	75.2 (7.2) 76.9 (6.2)	36.3 60.6	29.0 (1.5) 28.9 (0.9)	MMSE < 24	NPI	Pre-AD: higher sleep disturbances.
Palmer et al. [92]	MCI HS	69 72	75.7 (7.7) 71.8 (7.7)	60.9 58.3	25.6 (2.9) 28.6 (1.4)	Petersen	SCADS	MCI: 21.7% poor sleep quality. HS: 15.3% poor sleep quality.
Peters et al. [93]	MCI	193	67 (11.4)	53	26.3	DSM-IV-TR	NPI	39.9% sleep disturbance.
Pocnet et al. [65]	HS MCI AD	64 46 54	66 73 79	54.7 65.2 72.2	T1,2 = 29/29 T1,2 = 28/28 T1,2 = 24/23	Winblad NINCDS–ADRDA	NPI	Follow-up (2 years) MCI: increased sleep disturbances.
Reijs et al. [94]	MCI	353	70.6 (6.9)	59.0	26.2 (2.9)	NINCDS–ADRDA	NPI	22% sleep disturbances.
Rozzini et al. [95]	MCI	13	71.8 (7.4)	55	27.6 (1.9)	Artero	NPI	NPI: 0.5 (1.2)
Scaricamazza et al. [66]	MCI AD	20 45	69.7 (6.2) 66.4 (9.2)	60 64.4	26.8 (1.6) 22.4 (1.2)	Petersen NINCDS–ADRDA	NPI	AD: higher sleep disturbances.
Shin et al. [67]	AD	63	74.8 (6.1)	73	16.7 (4.4)	NINCDS–ADRDA	PSQI	PSQI TOT: 5.4 (3.9) SE (%): 87.2 (11.7) TIB (min): 510 (93.5)
Sun et al. [97]	aMCI HS	50 38	68.8 (5.9) 68.7 (5.5)	66.0 63.1	26.6 (1.3) 28.5 (1.1)	Petersen	PSQI ISI	PSQI aMCI: higher poor sleep quality. ISI aMCI: higher severe insomnia.
Tadokoro et al. [68]	HS MCI AD	22 20 21	74.0 (7.8) 77.6 (6.5) 75.5 (6.2)	59.1 50.0 61.9	29.0 (1.1) 26.9 (2.3) 20.7 (6.6)	DSM 5	PSQI	AD: higher poor sleep quality than MCI and HS.
Tuna et al. [98]	MCI	56	69.7 (4.0)	58.9	7	RCS < 8	PSQI	53.6% poor sleep quality.
Wams et al. [101]	HS aMCI	18 8	73.8 (4.6) 77.1 (4.0)	46.1 50	MMSE ≥ 27 26 < MMSE < 20	NINCDS–ADRDA	PSQI JSQ	No difference between HS and aMCI has been found.
Westerberg et al. [102]	aMCI HS	10 10	71.1 72.5	80 70	27.8 29.3	Petersen	PSQI Karolinska Sleep Diary	No difference between HS and aMCI has been found.
Westerberg et al. [103]	HS aMCI	16 8	72.7 (5.1) 75.6 (7.2)	- -	28.4 (0.4) 27.3 (0.6)	Petersen	PSQI	No difference between HS and aMCI has been found.
Xie et al. [107]	MCI HS	479 734	60–80+ 60–80+	61.4 49.3	- -	Portet Johar	AIS	MCI: higher SL, TST, WASO, early awakenings and sleep disturbances.
Yatawara et al. [69]	MCI AD	38 158	64.4 (9.1) 69.6 (9.6)	47.3 46.8	27.3 (1.7) 21.1 (4.7)	Petersen NIA-AA	NPI	AD: 40% sleep disturbances. MCI: 31% sleep disturbances.
Yu et al. [99]	HS MCI	48 48	68.1 (5.6) 67.9 (4.9)	66.7 83.3	MoCA: 26.2 (3.5) 24.2 (3.6)	Petersen	PSQI	MCI: higher poor sleep quality.
Zhou et al. [70]	AD HS	84 92	66.5 (7.0) 69.0 (8.2)	66.7 56.5	18.1 (3.2) 28.7 (1.8)	NINCDS–ADRDA	PSQI NPI	PSQI AD: higher poor sleep quality. NPI AD: higher sleep disturbances.
Wang et al. [109]	HS MCI	804 151	66.6 (5.1) 68.7 (5.8)	49 76.4	- - 23.6 (5.4)	MMSE < 17 Cui	Interview	MCI: Higher SD than HS.

SD = standard deviation; F = female; ES = standard error; HS = healthy subject; MCI = mild cognitive impairment; aMCI = amnestic mild cognitive impairment; naMCI = non-amnestic mild cognitive impairment; Pre-AD = preclinical Alzheimer’s disease; AD = Alzheimer’s disease; MMSE = Mini Mental State Examination; MoCA = Montreal Cognitive Assessment; CDR = Clinical Dementia Rating Scale; RCS = Rapid Cognitive Screen; T1,2 = Time 1 and Time 2; DSM = Diagnostic and Statistical Manual of Mental Disorders; NIA–AA = National Institute on Aging–Alzheimer’s Association; NINCDS = National Institute of Neurological and Communicative Diseases and Stroke; NINCDS–ADRDA = National Institute of Neurological and Communicative Diseases and Stroke–Alzheimer’s Disease and Related Disorders Association; PSQI = Pittsburgh Sleep Quality Index; NPI = Neuropsychiatric Inventory; ISI = Insomnia Severity Index; MEQ = Morningness–Eveningness Questionnaire; AIS = Athens Insomnia Scale; JSS = Jenkins Sleep Questionnaire; SCADS = Sleep Continuity Scale in Alzheimer’s Disease; SMHSQ = St. Mary’s Hospital Sleep Questionnaire; TST = total sleep time; TIB = time in bed; SL = sleep latency; SD = sleep duration; SE = sleep efficiency.

**Table 6:** Sleep in pathological older age. Summary of all the studies that use polysomnography.

Authors	Group	N°	Age (SD)	(%F)	MMSE (SD)	Diagnostic Criteria	Measurements	Results
Carnicelli et al. [100]	MCI HS	19 11	69.8 (15.5) 69.2 (12.6)	47.3 45.5	25.3 (1.2) 29.3 (0.6)	Petersen	PSG	MCI: lower %REM.
De Gennaro et al. [46]	HS AD	20 20	70.3 72.0	60 65	28.75 16.4	DSM IV NINCDS–ADRDA	PSG	AD: higher LREM1, LREM2. AD: lower %SWS.
Dykierek et al. [61]	AD HS	35 42	62.1 (8.9) 64.4 (7.5)	54.4 47.6	19.5 (5.2) 29.2 (1.0)	NINCDS–ADRDA	PSG	AD: lower SPT and REM density.
Gorgoni et al. [63]	AD aMCI HS	15 15 15	70.8 71.1 70.8	66.7 60.0 33.3	16.1 26.1 29.1	DSM IV NIA–AA Petersen	PSG	AD: lower %SWS than HS. No difference between MCI vs. HS and AD vs. MCI has been found.
Hita-Yañez et al. [104]	HS MCI	25 25	67.1 (5.3) 70.5 (6.8)	52 28	28.1 (1.3) 26.7 (2.4)	Petersen	PSG	MCI: higher AI SWS. MCI: lower %REM.
Hoch et al. [71]	AD HS	20 23	71.6 (4.9) 68.9 (3.8)	65 65.2	17.3 (5.7) 29.2 (0.9)	DSM III NINCDS–ADRDA	PSG	AD: higher SL, %NREM, and total recording time. AD: lower SE and %NREM2.
Hot et al. [72]	AD HS	14 14	76.7 (3.8) 76.7 (4.1)	50 57.1	24.8 (2.4) 29.4 (1.0)	NINCDS–ADRDA	PSG	AD: lower %SE.
Kundermann et al. [73]	AD	15	71.1 (ES: 1.8)	40	MMST: 21.7 (ES: 1.1)	NINCDS–ADRDA	PSG	SPT: 402.5 min (ES: 15.2) TST: 292.8 min (ES: 21.6) WASO:136.2 min (ES: 20.1) LREM: 124.1 min (ES: 19.4) SE: 59.5% (ES: 4.6) NREM 1: 8.6% (ES: 1.0) NREM 2: 30.6% (ES: 2.6) SWS: 13.1% (ES: 2.5) REM: 8.0% (ES: 1.2)
Liguori et al. [86]	AD HS	48 29	70.5 (7.6) 70.4 (9.9)	47.9 48.3	19.4 (5.3) 28.7 (1.1)	NINCDS–ADRDA	PSG	AD: higher LREM, WASO. and %NREM1. AD: lower %SE, %NREM3, %REM.
Liguori et al. [74]	AD HS	18 18	71.6 (3.9) 74.4 (2.8)	55.6 61.1	22.6 (1.3) 28.9 (2.1)	NINCDS–ADRDA	PSG	AD: higher SL, LREM, WASO, and NREM1. AD: lower TST, %SE, NREM3, and REM.
Liguori et al. [75]	AD	20	70.75 (8.1)	55	21.9 (5.2)	Biomarkers Diagnostic Criteria	PSG	TST (min): 368.7 (70.6) SE (%): 74.7 (13.7) LREM: 204.8(147.8) WASO: 130.8 (88.9) NREM1: 31.9 (10.2) NREM2: 49.4 (12.2) NREM3: 12.1 (8.2) REM: 6.5 (4.1)
Loewenstein et al. [76]	HS AD	8 9	61.9 60.3	37.5 33.3	-	DSM III	PSG	AD: lower delta waves.
Maestri et al. [105]	HS MCI AD	11 11 11	72.7 (5.9) 68.5 (7.0) 69.2 (12.6)	63.6 63.6 45.5	29.3 (1.0) 24.9 (1.2) 21.2 (0.8)	NINCDS–ADRDA	PSG	AD: higher NREM1 and NREM2 than HS. AD: higher NREM1 than MCI. AD: lower TST, NREM3, and REM than HS. MCI: lower REM than HS.
Montplaisir et al. [77]	HS AD	10 10	58.3 60.6	- -	29.3 (5.3) 20.6 (1.0)	NINCDS–ADRDA	PSG	AD: higher EEG Slowing Index. AD: lower K-complex, sleep spindles and REM.
Naismith et al. [106]	HS MCI	26 26	65.9 (9.8) 70.1 (9.9)	53.8 34.6	29.2 (1.1) 27.5 (2.1)	Petersen	PSG	MCI: lower LREM and WASO.
Prinz et al. [78]	HS AD	11 10	72.2 (10.6) 73.3 (11.4)	0 -	- 3.3 (6.5)	MMSE > 20	PSG	AD: higher wakefulness. AD: lower NREM3, NREM4, and REM
Rauchs et al. [79]	AD HS	14 14	76.9 (4.1) 75.1 (4.6)	64.3 64.3	24.9 (2.0) 29.4 (0.9)	NINCDS–ADRDA MMSE < 21	PSG	AD: lower sleep spindles activity.
Reynolds III et al. [80]	HS AD	24 22	69.5 (4.5) 70.9 (8.1)	66.7 68.1	- 16.5 (5.0)	DSM III	PSG	AD: lower TSA.
Sanchez-Espinosa et al. [96]	HS aMCI	21 21	67 (5.5) 69.8 (6.5)	47.6 28.6	28.3 (1.3) 26.7 (2.5)	Petersen	PSG	aMCI: higher AI SWS. aMCI: lower %REM.
Tsuno et al. [81]	AD	12	75.6 (10.7)	83.3	14.4 (5.7)	NINCDS–ADRDA	PSG	SL (min): 14.6 (5.9)
Westerberg et al. [103]	HS aMCI	16 8	72.7 (5.1) 75.6 (7.2)	- -	28.4 (0.4) 27.3 (0.6)	Petersen	PSG	aMCI: lower SWS.
Liguori et al. [84]	MCI mAD msAD HS	59 56 48 41	67.4 (8.4) 69.9 (7.3) 71.7 (7.2) 67.2 (8.6)	52.6 62.5 60.4 52	28.9 (1.5) 24.5 (1.9) 15.4 (3.2) 29.2 (0.9)	NIA–AA Albert	PSG	msAD: lower TST, %SE NREM3%, REM% than mAD, MCI, and HS. msAD: higher NREM1% than MCI and HS. msAD: higher TIB than HS, MCI, and mAD. mAD: higher TST, NREM3%, REM% than msAD but lower than MCI and HS. mAD: higher TIB than msAD. mAD: higher WASO, LREM and NREM1% than HS. mAD: higher SE% than msAD but lower than HS. MCI: higher TST, NREM3% than mAD and msAD. MCI: higher SE% than msAD but lower than HS. MCI: higher REM% than msAD and mAD but lower than HS. MCI: higher LREM and WASO than HS. MCI: higher NREM1% than HS but lower than mAD and msAD. MCI: lower TIB than msAD. HS: higher TST and NREM3% than mAD and msAD. HS: higher SE%, REM% and lower WASO, NREM%1 than MCI, mAD and msAD. HS: lower LREM than MCI and mAD.
Liu et al. [110]	HS sMCI pMCI	22 25 20	70.6 (6.0) 71.6 (5.2) 70.8 (4.3)	63.6 52 45	28.4 (1.7) 26.7 (2.0) 26.9 (1.6)	Petersen	PSG	HS: higher TST and SE% than sMCI and pMCI. HS: lower LREM than sMCI and pMCI.

SD = standard deviation; F = female; ES = standard error; HS = healthy subject; MCI = mild cognitive impairment; aMCI = amnestic mild cognitive impairment; naMCI = non-amnestic mild cognitive impairment; AD = Alzheimer’s disease; MMSE = Mini Mental State Examination; DSM = Diagnostic and Statistical Manual of Mental Disorders; NIA–AA = National Institute on Aging–Alzheimer’s Association; NINCDS–ADRDA = National Institute of Neurological and Communicative Diseases and Stroke–Alzheimer’s Disease and Related Disorders Association; PSG = polysomnography; EEG = electroencephalogram; TST = total sleep time; TSA = time spent asleep; TIB = time in bed; TBT = total bed time; SPT = sleep period time; SOL = sleep onset latency; SL = sleep latency; SD = sleep duration; SE = sleep efficiency; LNREM = nonREM latency; NREM = nonREM; LREM = REM latency; REM = rapid eye movement; LSWS = slow-wave sleep latency; SWS = sleep wave sleep; WASO = wake after sleep onset; W = wakefulness; AI = arousal index; mAD = mild Alzheimer’s disease; msAD = mild severe Alzheimer’s disease; sMCI = stable mild cognitive impairment, pMCI = progressive mild cognitive impairment.

**Table 7:** Sleep in pathological older age. Summary of all the studies that use actigraphy.

Authors	Group	N°	Age (SD)	(%F)	MMSE (SD)	Diagnostic Criteria	Measurement	Results
Alfini et al. [108]	HS MCI	153 26	71.8 (8.3) 77.3 (7.9)	67.3 50	- -	Albert	Actigraphy	MCI: lower SE% than HC.
Khou et al. [82]	HS AD	51 35	73.4 (6.5) 73.7 (7.8)	69 31	- -	NINCDS−ADRDA	Actigraphy	AD: higher TST and TIB.
Lee et al. [83]	AD HS	7 11	77.0 (4.3) 74.2 (5.2)	57.1 63.6	1 < CDR < 2 CDR: 0	NINCDS−ADRDA	Actigraphy	No difference between HS and AD has been found.
Liguori et al. [85]	HS AD	10 18	61.8(11.2) 71.0 (5.9)	40 55.5	- -	NIA–AA	Actigraphy	AD: lower SE% and higher SL.
Tadokoro et al. [68]	HS MCI AD	22 20 21	74.0 (7.8) 77.6 (6.5) 75.5 (6.2)	59.1 50.0 61.9	29.0 (1.1) 26.9 (2.3) 20.7 (6.6)	DSM 5	Actigraphy	AD: higher NREM than MCI and HS. AD: lower REM than MCI and HS. MCI: lower REM than HS.
Wams et al. [101]	HS aMCI	18 8	73.8 (4.6) 77.1 (4.0)	46.1 50	MMSE ≥ 27 26 < MMSE < 20	NINCDS−ADRDA	Actigraphy	No difference between HS and aMCI has been found.
Westerberg et al. [102]	aMCI HS	10 10	71.1 72.5	80 70	27.8 29.3	Petersen	Actigraphy	aMCI: higher variability. aMCI: start sleep later.
Wilckens et al. [59]	HS MCI	28 13	82.1 (7.2) 86.7 (10.2)	71.4 30.8	28.4 (1.4) 26.8 (2.0)	Albert	Actigraphy	No difference between HS and MCI has been found.

SD = standard deviation; F = female; ES = standard error; HS = healthy subject; MCI = mild cognitive impairment; aMCI = amnestic mild cognitive impairment; naMCI = non-amnestic mild cognitive impairment; AD = Alzheimer’s disease; MMSE = Mini Mental State Examination; CDR = Clinical Dementia Rating Scale; DSM = Diagnostic and Statistical Manual of Mental Disorders; NIA−AA = National Institute on Aging−Alzheimer’s Association; NINCDS−ADRDA = National Institute of Neurological and Communicative Diseases and Stroke–Alzheimer’s Disease and Related Disorders Association; TST = total sleep time; TIB = time in bed; SL = sleep latency; SE = sleep efficiency; NREM = nonREM; WASO = wake after sleep onset.

**Table 8:** Diurnal sleepiness in healthy older age. Summary of all the studies.

Authors	Group	N°	Age (SD)	(%F)	MMSE (SD)	Measurements	Results
Bernstein et al. [43]	HS	423	69.9 (7.6)	64.4	29.5	ESS	18% excessive diurnal sleepiness.
Ward et al. [127]	HS	84	73.2 (5.7)	48.8	29 (0.9)	ESS	ESS: 5.9 (3.7)

SD = standard deviation; F = female; HS = healthy subject; MMSE = Mini Mental State Examination Index; ESS = Epworth Sleepiness Scale.

**Table 9:** Diurnal sleepiness in pathological older age. Summary of all the studies.

Authors	Group	N°	Age (SD)	(%F)	MMSE (SD)	Diagnostic Criteria	Measurements	Results
Hita-Yañez et al. [88]	HS MCI	25 25	67.1 (5.3) 70.5 (6.8)	52 28	28.1 (1.3) 26.7 (2.4)	DSM 5 Petersen CDR ≤ 0.5	ESS	No difference between HS and MCI has been found.
Naismith et al. [106]	HS MCI	26 26	65.9 (9.8) 70.1 (9.9)	53.8 34.6	29.2 (1.1) 27.5 (2.1)	Petersen	ESS	MCI: higher diurnal sleepiness.
Sanchez-Espinosa et al. [96]	HS aMCI	21 21	67 (5.5) 69.8 (6.5)	47.6 28.6	28.3 (1.3) 26.7 (2.5)	Petersen	ESS	No difference between HS and aMCI has been found.
Sun et al. [97]	aMCI HS	50 38	68.8 (5.9) 68.7 (5.5)	66.0 63.1	26.6 (1.3) 28.5 (1.1)	Petersen	ESS	aMCI: higher diurnal sleepiness.
Tadokoro et al. [68]	HS MCI AD	22 20 21	74.0 (7.8) 77.6 (6.5) 75.5 (6.2)	59.1 50.0 61.9	29.0 (1.1) 26.9 (2.3) 20.7 (6.6)	DSM 5	ESS	No difference has been found.
Westerberg et al. [102]	aMCI HS	10 10	71.1 72.5	80 70	27.8 29.3	Petersen	ESS	No difference between HS and aMCI has been found.
Westerberg et al. [103]	HS aMCI	16 8	72.7 (5.1) 75.6 (7.2)	- -	28.4 (0.4) 27.3 (0.6)	Petersen	ESS	No difference between HS and aMCI has been found.
Zhou et al. [70]	AD HS	84 92	66.5 (7.0) 69.0 (8.2)	66.7 56.5	18.1 (3.2) 28.7 (1.8)	NINCDS–ADRDA	ESS	AD: higher diurnal sleepiness.

SD = standard deviation; F = female; HS = healthy subject; MCI = mild cognitive impairment; aMCI = amnestic mild cognitive impairment; AD = Alzheimer’s disease; MMSE = Mini Mental State Examination; CDR = Clinical Dementia Rating Scale; DSM = Diagnostic and Statistical Manual of Mental Disorders; NINCDS–ADRDA = National Institute of Neurological and Communicative Diseases and Stroke–Alzheimer’s Disease and Related Disorders Association; ESS = Epworth Sleepiness Scale.
